# Association of smoking status and nicotine dependence with multi-morbidity in China: A nationally representative crosssectional study

**DOI:** 10.18332/tid/166110

**Published:** 2023-06-16

**Authors:** Xinye Zou, Siyu Zou, Yi Guo, Di Peng, Hewei Min, Ruolin Zhang, Ruiwen Qin, Jianrong Mai, Yibo Wu, Xinying Sun

**Affiliations:** 1 Faculty of Education, University of Cambridge, Cambridge, United Kingdom; 2 School of Public Health, Peking University, Beijing, China; 3 School of Education, Qingdao Hengxing University of Science and Technology, Qingdao, China; 4 Department of Natural and Applied Science, Duke Kunshan University, Jiangsu, China; 5 College of Foreign Languages, Nanjing University of Aeronautics and Astronautics, Nanjing, China; 6 School of Nursing, Guangzhou Xinhua University, Guangzhou, China

**Keywords:** smoking status, nicotine dependence, multimorbidity, China, nationally representative

## Abstract

**INTRODUCTION:**

Multi-morbidity is a public health priority as it is associated with an increased risk of mortality and a substantial healthcare burden. Smoking is considered a predisposing factor for multi-morbidity, but evidence for an association between multi-morbidity and nicotine dependence is insufficient. This study aimed to explore the association between smoking status, nicotine dependence, and multi-morbidity in China.

**METHODS:**

We recruited 11031 Chinese citizens from 31 provinces in 2021 using a multistage stratified cluster sampling strategy to ensure the study population represented national population characteristics. The association between smoking status and multi-morbidity was analyzed using binary logistic regression and multinomial logit regression models. We then analyzed the associations between four kinds of smoking status (age at smoking initiation, cigarette consumption per day, smoking when ill in bed, and inability to control smoking in public places), nicotine dependence, and multi-morbidity among participants who were current smokers.

**RESULTS:**

Compared with non-smokers, the odds of multi-morbidity were higher among ex-smokers (adjusted odd ratio, AOR=1.40, 95% CI: 1.07–1.85). The risk of multi-morbidity was greater in participants who were underweight/overweight/obese (AOR=1.90; 95% CI: 1.60–2.26) compared with those who were normal weight. and also greater for drinkers (AOR=1.34; 95% CI: 1.09–1.63) than non-drinkers. Compared with children who began smoking at the age of <15 years, participants aged >18 years had a lower likelihood of multi-morbidity (AOR=0.52; 95% CI: 0.32–0.83). People who consumed ≥31 cigarettes per day (AOR=3.77; 95% CI: 1.47–9.68) and those who smoked when ill in bed (AOR=1.70; 95% CI: 1.10–2.64) were more likely to have multi-morbidity.

**CONCLUSIONS:**

Our findings show that smoking behavior, including initiation age, frequency of daily smoking, and still smoking during illness or in public, is a critical risk factor for multi-morbidity, especially when combined with alcohol consumption, physical inactivity, and abnormal weight (underweight, overweight, or obese). This highlights the crucial effect of smoking cessation in the prevention and control of multi-morbidity, especially in patients with three or more diseases. Implementing smoking and lifestyle interventions to promote health would both benefit adults and prevent the next generation from initiating habits that increase the risk of multi-morbidity.

## INTRODUCTION

Multi-morbidity is defined as the simultaneous presence of two or more chronic health disorders^1^. As the world population ages, the consequences of multi-morbidity are becoming more prevalent and severe. The number of individuals affected by multi-morbidity is increasing worldwide. A study in Scotland reported that 65% of patients aged ≥65 years and 80% of those ≥85 years had at least two chronic health conditions^2^. A study in southern China showed that >10% of 162464 community household residents had multi-morbidity^3^. Another study of 0.5 million middle-aged and elderly Chinese adults reported that 16% of the participants were multi-morbid^4^. It is widely recognized that multi-morbidity is associated with an increased risk of mortality^2^, decreased quality of life^5^, and substantial healthcare burden^6^. Multimorbidity further increases the financial burden and causes patients to be unable to pay out-of-pocket expenditures^7^. Thus, multi-morbidity has become a global public health priority requiring effective intervention. To design better prevention and management strategies, researchers need more data on the factors that influence multi-morbidity^8^.

A variety of factors are related to multi-morbidity, including sociodemographic factors^9,10^ and modifiable behaviors such as physical activity, obesity^11^, and smoking^12^. Smoking, in particular, has been identified as a predisposing factor for multi-morbidity in disease-free populations^12^. A similar conclusion was reached in a cross-sectional study of 109695 Canadians aged >12 years; people who smoked had a much higher prevalence of multi-morbidity than those who had never smoked^13^. In China, a large-scale prospective study among 0.5 million adults reported that people who smoke are more likely to develop cardiometabolic multi-morbidity^14^. However, most studies have been conducted in a limited geographical area or among an elderly population. Moreover, most research in China has focused on the relationship between smoking and the risk of specific disease comorbidity among hospital-based individuals.

Tobacco use represents a significant public health challenge worldwide. The Global Burden of Disease Study 2019 indicates that tobacco smoking causes 8 million deaths every year^15^, making tobacco-related illness the primary cause of preventable and premature mortality^16^. A review concluded that smoking has an apparent relationship with five comorbid domains: psychiatric, cancer, cardiovascular, pulmonary, and HIV infection^17^. Moreover, the number of patients with multiple chronic diseases is expected to increase. However, few studies have focused on smoking as a risk factor for multi-morbidity. Existing studies on multi-morbidity often do not investigate smoking behaviour in sufficient detail. Most researchers simply divide the population into smokers and non-smokers without further research on the association of multi-morbidity with tobacco use characteristics and dependence. A systematic review showed that most studies that diagnosed tobacco use status and dependence were conducted in developed countries and focused on a relationship with a singular disease domain^17^. Therefore, there is a need for detailed measures of smoking status among people with multi-morbidity. Surveys to explore the association of detailed smoking status with multi-morbidity are especially needed in developing countries, where people have a higher proportion of unhealthy smoking habits and a higher prevalence of multi-morbidity. Furthermore, most studies were conducted on people aged >30 years, and evidence is sparse regarding multi-morbidity and smoking among the Chinese general population.

This large, nationally representative, cross-sectional study investigated the relationship between smoking and multi-morbidity to estimate the different risks of multi-morbidity among different states of tobacco-smoking behavior. We hope to ascertain the specific relationship between smoking and multiple diseases and provide evidence to improve future interventions in multi-morbidity^18^.

## METHODS

### Study design and population

This is a nationwide cross-sectional study on the Chinese population aged ≥18 years^19^. Multistage, stratified cluster sampling was used to recruit participants from 120 cities across China’s 31 provinces from 10 July to 15 September 2021. In the first stage, 120 cities were selected: one capital city and one non-capital city in every province, autonomous region, and municipality. Based on sex, age, and region strata, 100 samples were selected from 120 cluster cities with a probability proportional to the population size. A unique web link for the electronic questionnaire was distributed face-to-face by trained investigators to voluntary participants in each city where the survey was conducted. Participation in the study was voluntary and all respondents provided informed consent. The interviewers were invited to a community healthcare center or community neighborhood committee to complete the questionnaire. After questionnaire collection, the quality check was conducted by two researchers independently. The screening criteria to determine high quality or valid questionnaire were as follows: 1) passed the logic check questions (Supplementary file); 2) speeder check to eliminate those with response time of fewer than 240 seconds; 3) deleted incomplete or repeated questionnaires; and 4) deleted questionnaires with all selected options being the same or having regularity. In total, 11031 valid questionnaires of high quality with signed informed consent were obtained. The China Family Health Index (CFHI) survey questionnaire includes the following modules: demographics, a short form of the Family Health Scale, and a 10-item Big Five inventory.

### Smoking status

The participants were asked about their current smoking status, which was classified as non-smoker, current smoker, or ex-smoker. For those who were current smokers, we asked them further: ‘How many cigarettes do you smoke per day?’, ‘What age did you first smoke?’, and ‘Do you have difficulty controlling your need to smoke in many non-smoking places?’. The data collected were classified as follows: age at first smoking (≤15, 16–18, and ≥19 years), cigarettes per day (≤10, 11–20, 21–30, and ≥31), smoking when ill in bed (no, yes), and smoking in public places (no, yes). Nicotine dependence was then assessed using the Fagerström test for nicotine dependence (FTND)^20^. The FTND is a standard 6-item instrument that measures the degree of physical addiction to nicotine, including the quantity of cigarette consumption, the compulsion to use, and the degree of physical dependence. Supplementary file Table 1 shows the detailed six questions and value meaning. Total score of FTND ranges from 0 to 10, with higher scores indicating higher levels of nicotine dependence. Depending on the FTND total score, nicotine dependence is categorized as very low (0–2), low (3–4), medium (5), high (6–7), and very high (8–10 points) level of dependence on nicotine. The FTND has been shown to be a reliable and valid measure of nicotine dependence^21^. In this study, FTND demonstrated good internal consistency with Cronbach’s coefficient α = 0.661.

**Table 1:** Baseline characteristics of participants, stratified by smoking status, China 2021 (N=11031)

*Characteristics*	*Overall (N=11031) n (%)*	*Smoking status*			*p^a^*
		*Non-smoker (N=8845) n (%)*	*Current smoker (N=1399) n (%)*	*Ex-smoker (N=787) n (%)*	
**Age** (years)					<0.001
≤30	4665 (42)	4212 (48)	334 (24)	119 (15)	
31–45	3001 (27)	2357 (27)	467 (33)	177 (22)	
46–60	2218 (20)	1523 (17)	433 (31)	262 (33)	
>60	1147 (10)	753 (8.5)	165 (12)	229 (29)	
**Sex**					<0.001
Female	5998 (54)	5787 (65)	114 (8.1)	97 (12)	
Male	5033 (46)	3058 (35)	1285 (92)	690 (88)	
**Multi-morbidity**					<0.001
Yes	646 (5.9)	405 (4.6)	128 (9.1)	113 (14)	
No	10385 (94)	8440 (95)	1271 (91)	674 (86)	
**Number of diseases**					<0.001
0	9084 (82)	7584 (86)	1041 (74)	459 (58)	
1	1301 (12)	856 (9.7)	230 (16)	215 (27)	
2	448 (4.1)	290 (3.3)	89 (6.4)	69 (8.8)	
≥3	198 (1.8)	115 (1.3)	39 (2.8)	44 (5.6)	
**Residence**					<0.001
Rural	3023 (27)	2351 (27)	436 (31)	236 (30)	
Urban	8008 (73)	6494 (73)	963 (69)	551 (70)	
**Monthly average income** (RMB)					0.400
≤3000	3246 (29)	2623 (30)	385 (28)	238 (30)	
3001–4500	2344 (21)	1897 (21)	297 (21)	150 (19)	
4501–7500	2981 (27)	2368 (27)	388 (28)	225 (29)	
>7500	2460 (22)	1957 (22)	329 (24)	174 (22)	
**Education level**					<0.001
Primary school/no formal education	1127 (10)	826 (9.3)	178 (13)	123 (16)	
Middle school	1439 (13)	1044 (12)	253 (18)	142 (18)	
Secondary school	1978 (18)	1500 (17)	288 (21)	190 (24)	
College/university	5750 (52)	4836 (55)	620 (44)	294 (37)	
Graduate	737 (6.7)	639 (7.2)	60 (4.3)	38 (4.8)	
**Employment status**					<0.001
Employed	4637 (42)	3535 (40)	769 (55)	333 (42)	
Student	3314 (30)	3115 (35)	136 (9.7)	63 (8.0)	
Unemployed	2196 (20)	1596 (18)	384 (27)	216 (27)	
Retired	884 (8.0)	599 (6.8)	110 (7.9)	175 (22)	
**Insurance**					<0.001
No	2299 (21)	1922 (22)	249 (18)	128 (16)	
Yes	8732 (79)	6923 (78)	1150 (82)	659 (84)	
**Ethnicity**					0.005
Han	10386 (94)	8337 (94)	1295 (93)	754 (96)	
Minority	645 (5.8)	508 (5.7)	104 (7.4)	33 (4.2)	
**Alcohol consumption**					<0.001
Non-drinker	6578 (60)	6029 (68)	252 (18)	297 (38)	
Drinker	4453 (40)	2816 (32)	1147 (82)	490 (62)	
**Regular physical activity**					0.300
Active	4027 (37)	3603 (41)	573 (41)	342 (43)	
Inactive	7004 (63)	5242 (59)	826 (59)	445 (57)	
**BMI**					<0.001
Normal	6758 (61)	5555 (63)	726 (52)	477 (61)	
Underweight/overweight/obese	4273 (39)	3290 (37)	673 (48)	310 (39)	

a Pearson's chi-squared test.

BMI: body mass index (kg/m^2^). RMB: 1000 Chinese Renminbi about US$ 140.

### Multi-morbidity

The primary outcome measure of the study was multi-morbidity. A systematic review of definitions and measurements of multi-morbidity found that multi-morbidity was commonly defined as the presence of multiple diseases or conditions, with a cutoff of two or more^1^. In accordance with previous studies, multi-morbidity was defined as the concurrence of two or more chronic conditions in the same individual. The diseases were selected based on insights from the 25 leading causes of DALYs in China^22^, the morbidities recommended as the core for any multi-morbidity measure by a UK-based study^2^, and the data available in CFHI datasets. The variable used for the self-reported diagnosis was the question: ‘Are you currently diagnosed with any of the following diseases?’ followed by a list of 20 diseases: hypertension, coronary heart disease, stroke, thrombus, diabetes, Alzheimer’s disease, Parkinson’s disease, viral hepatitis, neoplasms, chronic enteritis, chronic gastritis, emotional disorders, asthma, dyslipidemia, chronic obstructive pulmonary disease, chronic kidney disease, fatty liver disease, thyroid disease, osteoporosis, and lumbar disc herniation. Participants who answered ‘yes’ to the question were classified as having a self-reported chronic condition.

### Covariates

This study collected information on the following covariates: demographic factors including age (≤30, 31–45, 46–60, and ≥60 years), sex (female, male), and residence (rural, urban); socioeconomic factors including monthly average income (≤3000, 3001– 4500, 4501–7500, and ≥7500 RMB; 1000 Chinese Renminbi about US$140), education level (primary school/no formal education, middle school, secondary school, college/university, or graduate), employment status (employed, student, unemployed, or retired), and insurance (no, yes); health behaviors including alcohol consumption (non-drinker or, drinker), and physical activity (active, inactive); and anthropometric indicators including body mass index (BMI normal range: 18.5–24.0 kg/m^2^, or underweight/overweight/obese). In detail, monthly average income in the household was classified into quartiles (Q1: ≤3000; Q2: 3001–4500; Q3: 4501–7500; and Q4: >7500 RMB) with approximately the same number of people in each group. Alcohol consumption was measured by asking the participants: ‘Have you had any alcohol in the last 12 months?’. Participants who answered ‘Yes, within the last 30 days’ or ‘Yes, before the last 30 days’ were classified as ‘drinkers’, and those who answered ‘No’ were classified as ‘non-drinkers’. Physical activity was estimated by summing the metabolic equivalent task (MET) hours per week (h/week) spent based on the activities from the International Physical Activity Questionnaire (IPAQ). Moderate-to-vigorous activities included bodybuilding, walking, swimming, cycling, exercise with sports equipment, and other aerobic exercises (Supplementary file Table S2). The details of the measurements have been described elsewhere^23^. We grouped physical activity into active and inactive categories based on the median of total physical activity in all participants (<20.3 MET-h/week). BMI (kg/m^2^) was classified into normal from 18.5 to 24.0 kg/m^2^, otherwise, it was defined as abnormal (underweight <18.5, overweight 24.0 to <28.0, or obese ≥28.0).

### Statistical analysis

Frequencies, percentages, and cross-tabulations are reported for the descriptive analyses. The prevalence of smoking status and the number of chronic diseases (none, 1, 2, or ≥3 diseases) in each population were calculated using percentages and 95% confidence intervals (CIs) against the total population. Differences between different variables and smoking status and multi-morbidity were compared using the chi-squared test.

A two-stage statistical analysis was undertaken. First, we conducted multivariable logistic regression analysis to examine whether smoking status (non-smoker, current smoker, or ex-smoker) is associated with the presence of multi-morbidity (yes, no). Two nested logistic models were fitted: 1) unadjusted; and 2) adjusted for various demographic (age, sex, and residence) and socioeconomic factors (monthly income, education level, employment, and health insurance), lifestyle factors (physical activity, alcohol consumption, and BMI). Second, the outcomes were divided into four categories: no disease, 1, 2, and ≥3 diseases. Multinomial or ordinal logit regression model was utilized to examine the association between smoking status and different number of chronic diseases with ‘no disease’ as the reference group. We conducted an overall test of proportional odds assumption. If p<0.05, multinomial logistic regression was appropriate to be adopted; otherwise, ordinal logistic regression would be adopted. Results are reported as adjusted odds ratios (AORs) and 95% CI.

We then used a multivariable logistic regression model of participants who were current smokers to analyze the association of multi-morbidity (yes, no) with four kinds of tobacco-smoking status: age at first smoking, cigarette consumption per day, smoking when ill in bed, and inability to stop smoking in public places. The associations were tested in 2-tailed tests and considered statistically significant if p<0.05. All analyses were performed using Stata 16 MP (StataCorp, College Station, TX, USA) and R software.

## RESULTS

Table 1 presents the baseline characteristics of all the participants. Among the 11031 participants, 5.9% (n=646) had multi-morbidities. The percentages of non-smokers, current smokers, and ex-smokers were 80.2%, 12.7%, and 7.1%, respectively. A greater proportion of the participants were aged ≤30 years (42%), had insurance (79%), lived in urban areas (73%), were physically active (63%), and had a normal BMI (61%). When stratified by smoking status, demographic, socioeconomic, and behavior factors were all statistically significant among the different smoking groups, except for income and regular physical activity (Table 1).

Table 2 presents the smoking status among people with different number of diseases. The percentages of 1 disease, 2 diseases, and ≥3 diseases were 12%, 4.1%, and 1.8%, respectively. Among 1399 current smokers, 57% started smoking at age <18 years, 27% still smoked when ill in bed, and 25% could not control smoking in public places.

**Table 2:** Prevalence of multi-morbidity by demographic and socioeconomic characteristics, lifestyle factors and smoking status, China 2021 (N=11031)

*Characteristics*	*Overall (N=11031) n (%)*	*Number of diseases*				*p^a^*
		*None (N=9084) n (%)*	*1 (N=1301) n (%)*	*2 (N=448) n (%)*	*≥3 (N=198) n (%)*	
**Age** (years)						<0.001
≤30	4665 (42)	4465 (49)	156 (12)	35 (7.8)	9 (4.5)	
31–45	3001 (27)	2638 (29)	267 (21)	64 (14)	32 (16)	
46–60	2218 (20)	1536 (17)	487 (37)	146 (33)	49 (25)	
>60	1147 (10)	445 (4.9)	391 (30)	203 (45)	108 (55)	
**Sex**						<0.001
Female	5998 (54)	5087 (56)	617 (47)	204 (46)	90 (45)	
Male	5033 (46)	3997 (44)	684 (53)	244 (54)	108 (55)	
**Residence**						0.032
Rural	3023 (27)	2448 (27)	367 (28)	144 (32)	64 (32)	
Urban	8008 (73)	6636 (73)	934 (72)	304 (68)	134 (68)	
**Monthly average income** (RMB)						<0.001
≤3000	3246 (29)	2605 (29)	413 (32)	164 (37)	64 (32)	
3001–4500	2344 (21)	1932 (21)	271 (21)	99 (22)	42 (21)	
4501–7500	2981 (27)	2448 (27)	354 (27)	114 (25)	65 (33)	
>7500	2460 (22)	2099 (23)	263 (20)	71 (16)	27 (14)	
**Education level**						<0.001
Primary school/no formal education	1127 (10)	659 (7.3)	284 (22)	130 (29)	54 (27)	
Middle school	1439 (13)	1078 (12)	230 (18)	90 (20)	41 (21)	
Secondary school	1978 (18)	1588 (17)	270 (21)	81 (18)	39 (20)	
College/university	5750 (52)	5109 (56)	460 (35)	128 (29)	53 (27)	
Graduate	737 (6.7)	650 (7.2)	57 (4.4)	19 (4.2)	11 (5.6)	
**Employment status**						<0.001
Employed	4637 (42)	3915 (43)	527 (41)	144 (32)	51 (26)	
Student	3314 (30)	3174 (35)	110 (8.5)	21 (4.7)	9 (4.5)	
Unemployed	2196 (20)	1591 (18)	378 (29)	153 (34)	74 (37)	
Retired	884 (8.0)	404 (4.4)	286 (22)	130 (29)	64 (32)	
**Insurance**						<0.001
No						
Yes	8732 (79)	7054 (78)	1104 (85)	400 (89)	174 (88)	
**Ethnicity**						0.600
Han	10386 (94)	8556 (94)	1220 (94)	420 (94)	190 (96)	
Minority	645 (5.8)	528 (5.8)	81 (6.2)	28 (6.2)	8 (4.0)	
**Smoking**						<0.001
Non-smoker	8845 (80)	7584 (83)	856 (66)	290 (65)	115 (58)	
Current smoker	1399 (13)	1041 (11)	230 (18)	89 (20)	39 (20)	
Ex-smoker	787 (7.1)	459 (5.1)	215 (17)	69 (15)	44 (22)	
**Alcohol consumption**						<0.001
Non-drinker	6578 (60)	5527 (61)	702 (54)	247 (55)	102 (52)	
Drinker	4453 (40)	3557 (39)	599 (46)	201 (45)	96 (48)	
**Regular physical activity**						<0.001
Active	4518 (41)	3902 (43)	428 (33)	130 (29)	58 (29)	
Inactive	6513 (59)	5182 (57)	873 (67)	318 (71)	140 (71)	
**BMI**						<0.001
Normal	6758 (61)	5767 (63)	694 (53)	204 (46)	93 (47)	
Underweight/overweight/obese	4273 (39)	3317 (37)	607 (47)	244 (54)	105 (53)	
**Age started smoking**^b^ (years)						0.200
≤15	371 (27)	263 (25)	65 (28)	29 (33)	14 (36)	
16–18	421 (30)	314 (30)	74 (32)	20 (22)	13 (33)	
≥19	607 (43)	464 (45)	91 (40)	40 (45)	12 (31)	
**Cigarettes per day** ^b^						0.003
≤10	924 (66)	714 (69)	138 (60)	47 (53)	25 (64)	
11–20	350 (25)	248 (24)	64 (28)	30 (34)	8 (21)	
21–30	91 (6.5)	63 (6.1)	17 (7.4)	8 (9.0)	3 (7.7)	
≥31	34 (2.4)	16 (1.5)	11 (4.8)	4 (4.5)	3 (7.7)	
**Smoking when ill in bed** ^b^						<0.001
No	1104 (79)	848 (81)	168 (73)	67 (75)	21 (54)	
Yes	295 (21)	193 (19)	62 (27)	22 (25)	18 (46)	
**Cannot stop smoking in public places** ^b^						<0.001
No	1054 (75)	815 (78)	154 (67)	56 (63)	29 (74)	
Yes	345 (25)	226 (22)	76 (33)	33 (37)	10 (26)	

a Pearson’s chi-squared test.

b Among current smokers.

BMI: body mass index (kg/m^2^). RMB: 1000 Chinese Renminbi about US$ 140.

A significant association was observed between smoking status and multi-morbidity. Compared with non-smokers, the odds of having multi-morbidity were higher among ex-smokers (AOR=1.40; 95% CI: 1.07–1.85). The association with multi-morbidity was greater in participants aged >30 years than in those ≤30 years and slightly greater for insured versus uninsured participants. Urban dwellers (AOR=1.27; 95% CI: 1.02–1.57) and unemployed participants (AOR=1.36; 95% CI: 1.04–1.77) were more likely to be multi-morbid. An income of >7500 RMB (AOR=0.71; 95% CI: 0.53–0.95) was a protective factor for multi-morbidity. Being underweight/overweight/obese (AOR=1.90; 95% CI: 1.60–2.26), and a drinker (AOR=1.34; 95% CI: 1.09–1.63) were risk factors for multi-morbidity (Table 3).

**Table 3:** Association between smoking status and multi-morbidity, China 2021 (N=11031)

*Characteristics*	*Unadjusted model*		*Adjusted model^a^*	
	*OR (95% CI)*	*p*	*AOR (95% CI)*	*p*
**Smoking status**				
Non-smoker (Ref.)	1		1	
Current smoker	2.10 (1.71–2.58)	<0.001	1.27 (0.97–1.66)	0.084
Ex-smoker	3.49 (2.80–4.37)	<0.001	1.40 (1.07–1.85)	0.015
**Age** (years)				
≤30 (Ref.)	1		1	
31–45	3.47 (2.42–4.97)	<0.001	2.29 (1.47–3.57)	<0.001
46–60	10.12 (7.27–14.10)	<0.001	5.79 (3.78–8.88)	<0.001
>60	39.07 (28.25–54.03)	<0.001	19.42 (12.19–30.94)	<0.001
**Sex**				
Female (Ref.)	1		1	
Male	1.46 (1.24–1.71)	<0.001	1.03 (0.83–1.28)	0.775
**Residence**				
Rural (Ref.)	1		1	
Urban	0.78 (0.66–0.93)	0.005	1.27 (1.02–1.57)	0.031
**Monthly average income** (RMB)				
≤3000 (Ref.)	1		1	
3001–4500	0.85 (0.68–1.05)	0.134	0.93 (0.73–1.18)	0.545
4501–7500	0.85 (0.69–1.04)	0.104	1.03 (0.81–1.30)	0.833
>7500	0.55 (0.43–0.70)	<0.001	0.71 (0.53–0.95)	0.021
**Education level**				
Primary school/no formal education (Ref.)	1		1	
Middle school	0.51 (0.40–0.65)	<0.001	0.90 (0.68–1.18)	0.442
Secondary school	0.33 (0.26–0.42)	<0.001	0.83 (0.62–1.12)	0.221
College/university	0.17 (0.13–0.21)	<0.001	0.75 (0.56–1.02)	0.068
Graduate	0.22 (0.15–0.32)	<0.001	1.21 (0.75–1.97)	0.433
**Employment status**				
Employed (Ref.)	1		1	
Student	0.21 (0.14–0.31)	<0.001	0.67 (0.41–1.09)	0.109
Unemployed	2.63 (2.15–3.20)	<0.001	1.36 (1.04–1.77)	0.026
Retired	6.40 (5.17–7.94)	<0.001	1.33 (0.98–1.81)	0.066
**Insurance**				
No (Ref.)	1		1	
Yes	2.18 (1.70–2.79)	<0.001	1.40 (1.06–1.84)	0.016
**Ethnicity**				
Han (Ref.)	1		1	
Minority	0.95 (0.67–1.34)	0.759	0.99 (0.68–1.44)	0.958
**Alcohol consumption**				
Non-drinker (Ref.)	1		1	
Drinker	1.28 (1.09–1.50)	0.003	1.34 (1.09–1.63)	0.004
**Regular physical activity**				
Active (Ref.)	1		1	
Inactive	1.74 (1.46–2.07)	<0.001	1.13 (0.93–1.37)	0.216
**BMI**				
Normal (Ref.)	1		1	
Underweight/overweight/obese	1.93 (1.65–2.27)	<0.001	1.90 (1.60–2.26)	<0.001

a AOR: adjusted odds ratio; adjusted for age, sex, residence, income, education level, employment, insurance, and ethnicity, alcohol consumption, physical activity, and BMI (body mass index). Dependent variable: multi-morbidity (0=no multi-morbidity; 1=multi-morbidity).

Proportional odds assumption was tested with p=0.30. Ordinal logistic regression analysis was performed to analyze the relationship between smoking status and the number of diseases. Using disease-free status as a reference, the effect of 1, 2, and ≥3 diseases were analyzed. The results showed that smoking status, smoking cessation, increasing age, and alcohol consumption were risk factors for disease. Compared to non-smokers, current smokers had a 1.25 times greater risk of being comorbid with two or more diseases (AOR=1.25; 95% CI: 1.05–1.49) and ex-smokers had nearly two times the likelihood of having ≥3 diseases (AOR=1.72; 95% CI: 1.43–2.07) (Table 4).

**Table 4:** Relationship of smoking status and number of diseases, by ordinal logistic regression, China 2021 (N=11031)

*Characteristics*	*Number of diseases*	
	*AOR (95% CI)*	*p*
**Smoking status**		
Non-smoker (Ref.)	1	
Current smoker	1.25 (1.05–1.49)	0.011
Ex-smoker	1.72 (1.43–2.07)	<0.001
**Age** (years)		
≤30 (Ref.)	1	
31–45	2.25 (1.79–2.83)	<0.001
46–60	6.27 (5.00–7.86)	<0.001
>60	18.72 (14.29–24.51)	<0.001
**Sex**		
Female (Ref.)	1	
Male	0.99 (0.87–1.14)	0.928
**Residence**		
Rural (Ref.)	1	
Urban	1.36 (1.18–1.56)	<0.001
**Monthly income** (RMB)		
≤3000 (Ref.)	1	
3001–4500	0.88 (0.75–1.03)	0.120
4501–7500	1.00 (0.86–1.17)	0.979
>7500	0.82 (0.69–0.97)	0.022
**Education level**		
Primary school/no formal education (Ref.)	1	
Middle school	0.79 (0.66–0.96)	0.017
Secondary school	0.77 (0.64–0.94)	0.010
College/university	0.64 (0.53–0.78)	<0.001
Graduate	0.78 (0.58–1.06)	0.118
**Employment status**		
Employed (Ref.)	1	
Student	0.74 (0.58–0.96)	0.022
Unemployed	1.16 (0.99–1.37)	0.073
Retired	1.33 (1.09–1.63)	0.005
**Insurance**		
No (Ref.)	1	
Yes	1.14 (0.97–1.33)	0.111
**Ethnicity**		
Han (Ref.)	1	
Minority	1.08 (0.86–1.36)	0.507
**Alcohol consumption in the last 12 months**		
Non-drinker (Ref.)	1	
Drinker	1.36 (1.20–1.54)	<0.001
**Regular physical activity**		
Active (Ref.)	1	
Inactive	1.16 (1.03–1.31)	0.014
**BMI**		
Normal (Ref.)	1	
Underweight/overweight/obese	1.70 (1.52–1.89)	<0.001

AOR: adjusted odds ratio; Model adjusted for age, sex, residence, income, education level, employment, insurance, ethnicity, alcohol consumption, physical activity, and BMI (body mass index).

**Figure 1:**
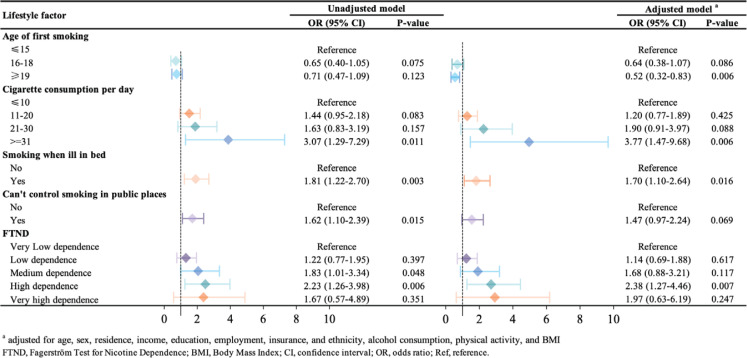
Association between smoking factors and multimorbidity, among current smoker (N=1399)

Figure 1 shows the association between detailed smoking status and multi-morbidity among current smokers. Compared to children who began smoking <15 years, those who first smoked at age ≥19 years had a lower likelihood of having multi-morbidity (AOR=0.52; 95% CI: 0.32–0.83). Compared to people who consumed ≤10 cigarettes per day, people who consumed ≥31 cigarettes were more likely to have multi-morbidity (AOR=3.77; 95% CI: 1.47–9.68). People who still smoked when ill in bed (AOR=1.70, 95% CI: 1.10–2.64) were more likely to have multimorbidity than those who did not. According to the FTND score, people who had high levels of nicotine dependence were more likely to have multi-morbidity, compared to those with very low levels of nicotine dependence (AOR=2.38; 95% CI: 1.27–4.46) (Figure 1).

## DISCUSSION

In this large-scale nationally representative study in China, 20% of the participants, aged mainly 30–60 years, had a smoking history. Compared with nonsmokers, ex-smokers had the highest risk of multimorbidity, followed by current smokers. Current smokers and those who quit smoking were more likely to have a higher number of chronic diseases. Participants who started smoking during childhood (aged <15 years), had greater cigarette consumption per day, smoked when ill in bed, and had high nicotine dependence were independently associated with a greater risk of multi-morbidity.

The relationship between current smokers and multi-morbidity was positive and significant but attenuated when adjusted for socioeconomic status and lifestyle factors. This may be explained by the confounding effect of age and a potential association between smoking cessation and multi-morbidity, since an adverse effect of tobacco use on health was identified. Some evidence has been found for an association between smoking status and the risk of multi-morbidity. In a multicenter cohort study across 10 European countries, pre-diagnostic unhealthy lifestyle behaviors, including smoking, were strongly associated with the risks of developing chronic non-communicable diseases, cancer multimorbidity, and cardiometabolic diseases^24^. Data from the China Kadoorie Biobank study revealed adverse effects from high-risk lifestyle factors (e.g. current smoking and quitting because of illness) on the risk of cardiometabolic multi-morbidity and death among 0.5 million adults aged 30–79 years^14^. In an longitudinal study of ageing conducted in the UK, physical inactivity combined with smoking increased the risk of multi-morbidity twice as much as the former alone, and combinations of unhealthy lifestyle factors significantly increased the multi-morbidity hazard^25^. Women aged 45–50 years who were current smokers were found to have increased odds of diabetes, heart disease, and stroke multi-morbidities in the Australian Longitudinal Study on Women's Health^26^. Our findings are largely consistent with those of prior studies and found a further positive association between smoking history and a higher number of multi-morbidities. This highlights the crucial effect of smoking cessation in the prevention and control of multi-morbidity, especially in patients with ≥3 diseases.

Previously the relationship between smoking use and multi-morbidity has not been explored fully. This study goes beyond, as we explored nicotine dependence-related smoking factors as predictors of multi-morbidity among current smokers. Most current smokers in China began smoking younger than an age of 18 years, and those who began smoking earlier showed a greater risk of multi-morbidity than smokers who started at a later age. Moreover, about 25% of smokers could not stop smoking in public, and about 25% still smoked when ill in bed. People highly dependent on tobacco were more likely to have multi-morbidity, suggesting that treatment for nicotine dependence has the potential for preventing multi-morbidity. This finding is consistent with previous studies conducted in middle-income countries. For example, a large-scale study in Cuba showed that people who had smoked since childhood had difficulty quitting smoking and a higher risk of premature death^27^. Studies in developing countries have shown that smoking is the leading risk factor for death; 87% of deaths occur among current smokers^15^. Thus, reducing the prevalence of smoking is a global health concern. However, the relatively high smoking prevalence and low rate of cessation in our Chinese population highlight the need for effective policies and management strategies for smoking prevention and control of tobacco use. Moreover, measures should be taken at the policy, primary-health promotion, and education levels to reduce the number of underage smokers.

Our results, consistent with previous research^14,24-26^, suggest the association of unhealthy lifestyle with multi-morbidity, emphasizing the need to integrate comprehensive lifestyle interventions into multi-morbidity management and to improve clinical outcomes. Further research should be undertaken to investigate the dynamic trends in lifestyle-behavior changes to the first occurrence, types, and progress of multi-morbidity and mortality. Moreover, etiological pathways shared among multiple unhealthy-lifestyle factors leading to multi-morbidity need to be investigated further^28,29^. Our research indicates that sociodemographic characteristics are influencing factors on multi-morbidity and the number of diseases. People aged >30 years, living in urban areas, unemployed, with low income, and with insurance have a greater risk of multi-morbidity and more types of disease. The OR of multi-morbidity and the number of diseases increased gradually with age and was highest among people aged >60 years. These results are consistent with those of previous studies with regard to age^30-32^, residence^33,34^, occupational status^35,36^, income^37,38^, and insurance^39,40^. Further work is required to investigate the causes of the unequal distribution of multi-morbidity across the sociodemographic spectrum, especially among different socioeconomic groups. Eliminating health disparities remains a crucial issue in promoting better health.

### Strength and limitations

This large-scale national representative study examined the epidemiology of multi-morbidity and tobacco use across the whole population to explore their relationship in the Chinese population. Multi-morbidity was defined using an international definition and included 20 types of chronic diseases that are common in China. We also explored multiple types of tobacco-smoking behavior, including age at smoking initiation, daily cigarette consumption, smoking when ill in bed, and inability to control smoking in public places. Given the size and diversity of the study design, our findings can be generalized to the greater Chinese population.

This study has several limitations. First, this study suffers from the typical limitations of a cross-sectional design; thus, we were unable to establish a causal relationship between multi-morbidity and smoking. Although the study asked participants about their starting age of smoking, it did not ask the age of onset of different diseases. As a result, we could not determine the temporal relationship between smoking initiation and multi-morbidity in this cross-sectional study. Also, smoking cessation due to multi-morbidity may cause reverse causality; therefore, the risk of smoking might have been underestimated. Further longitudinal studies are needed to examine the unbiased effects of smoking on multi-morbidity. Second, self-reported questionnaires can be influenced by recall bias. The age at smoking initiation, cigarette consumption per day, chronic disease types, and other self-reported information may have been misreported. Because the social acceptability of nicotine dependence and multi-morbidity is low, smoking status and multimorbidity may be under-reported, suggesting that our findings might have underestimated the actual prevalence of smoking and multi-morbidity. Third, this study consisted mainly of young people aged ≤30 years; therefore, only a few participants had developed complex multi-morbidities. Counting multi-morbidity as the number of chronic diseases is sufficient to show a risk from smoking. Further research should explore the effect of different sources and severities of smoking exposure on the first occurrence, progress, and outcomes of various multi-morbidity patterns.

## CONCLUSIONS

This nationally representative large-scale study showed that smoking status and nicotine dependence, combined with unhealthy lifestyle behaviors (alcohol consumption, physical inactivity, and abnormal BMI), are crucial risk factors for multi-morbidity and an increased number of chronic diseases. Most current smokers in China began smoking before the age of 18 years, about 25% of smokers could not stop smoking in public areas, and about 20% still smoked when ill in bed. People highly dependent on tobacco were more likely to have multi-morbidity, suggesting that treatment for nicotine dependence can be a strategy to improve health. Our study demonstrates the potential of evidence-based tobacco control policy implementation and enforcement in helping manage multi-morbidity of people in China. This policy would both benefit adults and prevent the next generation from initiating smoking habits.

## Data Availability

All data generated or analyzed during this study are included in this published article and the Supplementary file.
