# Supplementary material for: Association of smoking status and nicotine dependence with multi-morbidity in China: A nationally representative crosssectional study
Source: Tob Induc Dis. 2023 Jun 16;21:81. doi: 10.18332/tid/166110 (PMC10273826; doi:10.18332/tid/166110)
Supplement: Supplementary file 1 [file TID-21-81-s1.pdf]

Supplemental material

Using Logic Check Questions (LCQs) to screen out inconsistent respondents.

a. Fill in "≤18 years old" in Question 16 "How old are you?" but choose "married family", "DINK family" or "single-family" in Question 3 "What is your family type?";

b. Fill in "≤18 years old" in Question 16 "How old are you?" but choose "married", "divorced" or "widowed" in Question 21 "What is your marital status?";

c. Fill in "≤18 years old" in Question 16 "How old are you?" but choose "spouse", "spouse's father" or "spouse's mother" in Question 25 "Who lived with you in the last two months?";

d. Fill in "≤18 years old" in Question 16 "How old are you?" but choose "Communist Party member" or "Communist Party probationary member" in Question 19 "What is your political status?";

e. Outliers of height, weight, and BMI are problematic and should be removed because they represent measurement errors, data entry or processing errors.

Supplemental Table S1. Fagerstrom Test for Nicotine Dependence (FTND)

Items	Question/description	Value meaning
Fagerstrom Test Waking First Cigarette Score	How soon after you wake up do you smoke your first cigarette?	3 = Within 5 minutes, 2 = 6 to 30 minutes, 1 = 31 to 60 minutes, 0 = after 60 minutes
Fagerstrom Test Difficulty Stop Public Smoking Score	Do you find it difficult to refrain from smoking in places where it is forbidden, e.g. in church, at the library, in the cinema?	1 = Yes, 0 = No
Fagerstrom Test Hardest Stop Cigarette Score	Which cigarette would you hate most to give up?	1 = The first in the morning, 0 = Any other
Fagerstrom Test Cigarettes Per Day Score	How many cigarettes per day do you smoke?	0 = 10 or less, 1 = 11 to 20, 2 = 21 to 30, 3 = 31 or more
Fagerstrom Test First Hours Smoking Score	Do you smoke more frequently during the first hours after waking than the rest of the day?	1 = Yes, 0 = No
Fagerstrom Test Impaired Health Smoking Score	Do you smoke when you are so ill that you are in bed most of the day?	1 = Yes, 0 = No
**Total score**	Range: 0-10<br>0 to 2: Very low level of dependence on nicotine; 3 to 4: Low level of dependence on nicotine; 5: Medium level of dependence on nicotine; 6 to 7: High level of dependence on nicotine; 8 to 10: Very high level of dependence on nicotine	0 = Very Low Dependence, 1 = Low Dependence, 2 = Medium Dependence, 3 = High Dependence, 4 = Very High Dependence

Supplemental Table S2. Physical activity types, MET values, codes and intensity categories*

Activity type	Intensity	MET	Codes*
Fitness (weight lifting, vigorous effort, et.al)	Moderate	6	2050
Walking, for pleasure, work break	Moderate	3.5	17250
Swimming‡	Vigorous	7.2	Mean of 18230, 18240, and 18310
Bicycle	Vigorous	7.5	1015
Exercise with sports equipment (e.g., treadmill ergometer/ trampoline)†	Vigorous	6.25	Mean of 02065 (9), 15700 (3.5)
Other aerobic exercise (e.g., table tennis/jogging)†	Moderate	5.5	Mean of 15660 (4), and 12020 (7)

MET: Metabolic equivalent of tasks.

* Based on the 2011 Compendium of Physical Activities: a second update of codes and MET values. Ainsworth BE, et al. Medicine and Science in Sports and Exercise, 2011;43(8):1575-1581.

† Assigned 1/2 weight in calculating the mean MET value because the connecting two items represent one type of activity.

‡ Assigned 1/3 weight in calculating the mean MET value because the connecting three items represent one type of activity.

© **2023 Zou X. et al.**
